# Draft Genome Sequence of *Brevibacillus* *reuszeri* Strain NIT02, Isolated from a Laundered Rental Cloth Hot Towel

**DOI:** 10.1128/genomeA.01353-17

**Published:** 2018-01-11

**Authors:** Ken-Ichi Sano, Asuka Anraku

**Affiliations:** a Department of Innovative Systems Engineering, Nippon Institute of Technology, Miyashiro, Saitama, Japan; b Graduate School of Environmental Symbiotic System Major, Nippon Institute of Technology, Miyashiro, Saitama, Japan

## Abstract

*Brevibacillus reuszeri* is a Gram-positive spore-forming bacterium. Here, we present the draft genome sequence of *Brevibacillus reuszeri* strain NIT02, which was isolated from a laundered rental cloth hot towel.

## GENOME ANNOUNCEMENT

Oshibori are moist hot hand towels that are used in most restaurants located in Japan and have recently been used in many restaurants all over the world. In Japan, cloth oshibori are widely distributed and rented to restaurants and bars and are laundered and reused after each use. The cloth rental oshibori are laundered and sterilized with sodium hypochlorite and then packed into nylon using a packing machine. It is known from experience that when several months have passed after sterilization, cloth oshibori can develop an unpleasant odor that is similar to that of a wet and dirty rag or an acidic or sweaty odor. Such malodors are generated from microbes surviving on the cloth (1) and are often described in Japan as having a “natto-like” smell. Natto is a Japanese food made of soybeans fermented by *Bacillus subtilis* natto.

To isolate microbes that cause such unpleasant odors in laundered oshibori, rental cloth oshibori that had been produced 3 months prior (provided by Makishokai, Saitama, Japan) were dipped in 100 mL of phosphate-buffered saline. After fluid in the oshibori was squeezed into a beaker, bacterial cell bodies were collected and grown on a standard method agar medium. Four *Bacillus* families were isolated, and three of them were identified as *Brevibacillus reuszeri*. It has been shown in a number of studies that the spores of *Bacillus* spp. can survive treatment with wet heat and a variety of oxidizing agents. To investigate whether these *Bacillus* families were the cause of the unpleasant odor from laundered oshibori, we identified the draft genome of one of them, *Brevibacillus reuszeri* strain NIT02.

Genomic DNA was extracted using NucleoSpin microbial DNA (Macherey & Nagel, Duren, Germany) according to the manufacturer’s protocol. A sequence library was prepared using a KAPA HyperPlus library preparation kit (Roche Nimblegen, Madison) with a FastGene adapter kit (Nippon Genetics, Tokyo, Japan). The library was sequenced by using an Illumina MiSeq platform, and low-quality reads were filtered. The filtered reads were assembled by SPAdes 3.10.1 (2), 7,127,729 bp within 197 contigs was obtained, and the *N*_50_ of the contigs was 259,300 bp. The G+C content of the genome was 46.92%. Gene prediction was carried out using Prokka 1.11 (3), and then all the predicted open reading frames (ORFs) were manually predicted by BLAST (4). A total of 6,774 ORFs, 121 tRNAs, and 10 Bacillaceae-1 RNA motifs were found in the draft genome.

### Accession number(s).

This whole-genome shotgun project has been deposited at DDBJ/EMBL/GenBank under the accession number BEXF00000000. The version described in this paper is version BEXF01000000.
